# Genetic analysis reveals the inconsistency of amorpha-4,11-diene synthase, a key enzyme in the artemisinin synthesis pathway, in asteraceae

**DOI:** 10.1186/s13020-023-00708-w

**Published:** 2023-01-11

**Authors:** Shiyu Chen, Baosheng Liao, Shuai Guo, Xiaofeng Shen, Ying Meng, Yu Liang, Jiang Xu, Shilin Chen

**Affiliations:** 1 Pharmacy College, Chengdu University of Traditional Chinese Medicine, Chengdu, 611137 China; 2 Institute of Chinese Materia Medica, China Academy of Chinese Medical Sciences, Beijing, 100700 China; 3 Key Laboratory of Quality Evaluation of Chinese Medicine of the Guangdong Provincial Medical Products Administration, the Second Clinical College, Guangzhou University of Chinese Medicine, Guangzhou, 510006 China; 4 Institute of Medicinal Plant Development, Chinese Academy of Medical Sciences, Beijing, 100193 China

**Keywords:** Artemisia annua L., Amorpha-4,11-diene synthase, Genetic analysis, Phylogenetic analysis, Asteraceae, Functional identification

## Abstract

**Background:**

Amorpha-4,11-diene synthase (ADS) is a key enzyme in the artemisinin biosynthetic pathway. ADS promotes the first step of artemisinin synthesis by cyclizing faresyl pyrophosphate to synthesize the sesquiterpene product amorpha-4,11-diene. Thanks to the continuous improvement of genomic information, its evolutionary trace can be analyzed in a genome view.

**Methods:**

Phylogenetic analysis was used to identify *ADS*-like genes in other Asteraceae. Gene structure and motif analysis was used to analyze the structural similarity of these identified genes. Heterologous expression and GC–MS analysis were performed to determine whether the functions of *ADS* and *Cna4666* are consistent. Validation of *ADS* genes evolutionary trajectories was achieved by selective pressure and synteny analysis.

**Result:**

In this study, we extracted 8 *ADS* genes from the *Artemisia annua* L. genome annotation and 121 *ADS* similar genes from the genomes of Artemisia annua L. and other plants in the Asteraceae, and further exploring their evolutionary relationship. Phylogenetic analysis showed that the genes most closely related to *ADS* genes were found in the genome of *Chrysanthemum nankingense.* Among them, the gene structure and motif composition of Cna4666 is very similar to ADS, we wondered whether it has the potential to synthesize amorpha-4,11-diene. Therefore, we extracted the products of recombinant *p0_ADS.1* and *Cna4666* proteins by HS-SPME combined with GC–MS analysis, the results indicate that *Cna4666* is an α-bisabolol synthase, which cannot synthesize amorpha-4,11-diene. Through synteny analysis, we did not find collinear blocks of *ADS* genes in the *Helianthus annuus* and *C. nankingense* genomes. Furthermore, Ka/Ks ratios indicated that the evolution of *ADS* genes from their similar genes principally underwent purifying selection, and there was a strong positive selection between *ADS* genes.

**Conclusions:**

This study proved that *ADS* is a multi-copy gene in *Artemisia annua* L., and they are not widely distributed in Asteraceae. The data will increase our understanding of the evolutionary selection pressure on *ADS* genes. The results suggest that *ADS* genes are subject to strong positive selection internally, and it is possible that they are a recently evolved gene in the Artemisia.

**Supplementary Information:**

The online version contains supplementary material available at 10.1186/s13020-023-00708-w.

## Background

Artemisinin is the most useful drug ingredient presently used to treat malaria. The combinational therapies based on artemisinin have been suggested by the World Health Organization (WHO) as the preferred choice of action for the treatment of malaria affected by Plasmodium since 2002 [1, 2]. Artemisinin has saved millions of lives from malaria since its discovery in 1972 [3]. To date, the source of artemisinin has been mainly extracted from *Artemisia annua* L [4].

Most of the synthesis pathway of artemisinin has been clearly explained and widely agreed upon, and seven related genes have been identified in the process from faresyl pyrophosphate (FPP) to artemisinin or arteannuin B (Additional file 4: Fig. S1). FPP is synthesized from isopentenyl pyrophosphate (IPP) and dimethylallyl pyrophosphate (DMAPP) produced by the intracellular mevalonate (MVA) pathway or the methylerythritol 4-phosphate (MEP) pathway of plastids through farnesyl pyrophosphate (FPS) [5]. FPP is cyclized by amorpha-4,11-diene synthase to form amorpha-4,11-diene (AD) [6]. Then, AD is oxidized by cytochrome P450 monooxygenase (CYP71AV1) and alcohol dehydrogenase 1 (ADH1), catalyzed successively to form artemisinic alcohol, artemisinic aldehyde, and artemisinic acid successively [7–9]; Artemisinic aldehyde is oxidized by double bond reductase (DBR2) to form dihydroartemisinic aldehyde [9], which is then catalyzed by ALDH1 to form dihydroartemisinic acid [10]. Artemisinic acid and dihydroartemisinic aldehyde form arteannuin B and artemisinin, respectively, in a non-enzymatic reaction in Glandular-secreting trichomes [11–13]. *ADS* is the first step gene in the synthesis pathway of artemisinin, and is a significant component in the artemisinin biosynthesis pathway.

Terpenes mainly exist as monoterpenes, sesquiterpenes or diterpenes. Amorpha-4,11-diene belongs to sesquiterpene, so ADS is a sesquiterpene synthase. Sesquiterpene synthase catalyzes reactions with seven possible precursors, such as (E,E)-famesol, (Z,E)-famesol, (E)-α-famesol, (E)-β-famesol, (3R,6E)-nerolidol, (3S,6E)-nerolidol, etc. The initial cyclization reactions of sesquiterpene synthases can be divided into 1,10-closure and 1,11-closure of farnesyl cation or 1,6-closure, 1,7-closure, 1,10-closure, 1,11-closure of nerolidyl cation. With subsequent cyclizations, it is often difficult to determine the sequence of ring closures [14]. For example, δ-cadinene synthase from cotton undergoes 1,10-cyclization before 1,6-closure in the cyclization reaction [15, 16]; *A. annua* has another sesquiterpene synthase named 2-epi-cedrol synthase, which forms 1,6- cyclization followed by 6,10 and 2,11-closure [17, 18]. In addition, there are sesquiterpene synthases that do not undergo cyclization and produce acyclic sesquiterpenes, such as (E)-β-farnesene synthase from *Artemisia annua *[19]. A review has been published summarizing the discovery and catalytic process of ADS, it also described the application of ADS in metabolic engineering and synthetic biology and what important role sesquiterpene synthases played in the evolutionary development of artemisinin [20]. Including the following information, there was 36% protein similarity between ADS and tobacco 5-epi-aristolochene synthase (TEAS) as well as showed 41% amino acid sequence similarity with cotton ( +)-δ-cadinene synthase. And by observing the by-products catalyzed in the enzymatic reaction, it was hypothesized that amorpha-4,11-diene synthase catalyzed the 1,6-closure first followed by 1,10-closure [21], which was later confirmed [22–26]. The turnover rate or affinity of CYP7AV1 for ADS by-products was low, so it was pointed out that CYP71AV1 has a great catalytic specificity for AD [27]. Melissa Salmon et al. studied ADS and another FPP-based enzyme, (E)-β-farnesene synthase (BFS), a cyclized sesquiterpene synthase and a linear sesquiterpene synthase. And this study revealed that the cyclization of ADS may be due to a dominant natural mutation Y402L, or it could also be argued that Y402L has a prospective status in the functional differentiation of sesquiterpene synthases in *A. annua* [19]. A previous study investigated ADS homologs in 13 Artemisia species that do not produce artemisinin by PCR and showed that two new proteins, ( +)-α-bisabolol synthase and koidzumiol synthase, were cloned in three Artemisia species using ADS primers [28].

Genomics has played an important role in research fields such as plant biological characteristics, analysis of bioanabolism pathway, and molecular assisted breeding [29–39]. More and more plant genomes have been published, and the unique characteristics of plant genomes are becoming clearer. In addition, intraspecific and interspecific comparison of genome sequences can be used to infer species evolution and analyze phenotype-genotype relationships, which provides solid genetic support for plant research. Recently, a new *A. annua* genome has been published, and the assembled genome has high integrity and accurate allelic typing [40]. Therefore, we selected two haplotype genomes of *A. annua* and combined them with some Asteraceae plant genomes published several years ago to study *ADS* genes, including *Cynara cardunculus* var. Scolymus genome, *Helianthus annuus* genome, *Lactuca saligna* genome, *Lactuca sativa* genome and *Chrysanthemum nankingense* genome [36, 41–43]*.*

As research on artemisinin and artemisinin biosynthesis genes has progressed, we know that artemisinin can be detected in many Artemisia species and that the expression of artemisinin biosynthetic genes, including ADS genes, can be detected [44–47]. Then the question of whether *ADS* genes are also widely present in Asteraceae has not been answered. The present study is based on the recently published chromosome-level genome of A. annua with the support of some other Asteraceae genomes. This study reveals for the first time the inconsistency of *ADS* gene in Asteraceae and investigates the evolution of *ADS* in Asteraceae.

## Material and method

### Identification and characterization of ADS genes and similar genes in other species

A three-stair analysis was operated to extracted *ADS* genes from the *Artemisia annua L.* database of genome. Genomic and RNA-seq data of *A. annua* were obtained from Global Pharmacopoeia Genome Database (GPGD, http://www.gpgenome.com/, accessed on 22 April 2022) [48]. The public reference *ADS* coding sequence were downloaded by accession code AF138959.1, AAF61439.1, AF327527.1, AAK15697.1, AF327526.1, AAK15696.1, AJ251751.1, CAB94691.1, AY006482.1, AAF98444.1, DQ241826.1, ABB51572.1, EF197888.1, ABM88787.1, FJ432667.1, ACL15394.1, HQ315833.1, ADU25497.1, JQ319661.1, AFA34434.1, KJ609176.1, AIC83777.1, KR445687.1, ALJ03212.1, LC106014.1, BAW34953.1, PKPP01006435.1, PWA56512.1, FJ613423.1 and ACM80358.1 from National Center for Biotechnology Information (NCBI) Nucleotide Search database(https://www.ncbi.nlm.nih.gov/nuccore/, accessed on 23 February 2022), as query sequences to blast against predictional genomic coding sequences and genome-wide sequence (identity ≥ 95%, query coverage ≥ 90%). Then, all genes were manually corrected in Apollo, according to the result of blast in transcriptome data with public reference *ADS* genes. In the end, only genes with complete gene structure and supported by transcriptome or full-length transcript data were retained, and incomplete prediction results were removed to finalize the position and structure of each *ADS* gene.

Genomic protein prediction results for five Asteraceae species, including *Lactuca sativa*, *Helianthus annuus*, *Lactuca saligna*, and *Cynara cardunculus* var. Scolymus, were downloaded from NCBI Genome Database (https://www.ncbi.nlm.nih. gov/genome, accessed on 25 April 2022) [41–43], and Chrysanthemum Genome Database (http://www.amwayabrc.com/download.htm, accessed on 25 April 2022) [36]. The transcriptome data of *Artemisia argyi* leaf were downloaded from the Sequence Read Archive (SRA) database of NCBI with the accession code PRJNA804653 and used the transcripts were assembled using Trinity without reference. After that, the Genomic protein prediction results of *Artemisia annua* L., *Chrysanthemum nankingense*, *Cynara cardunculus* var. scolymus, *Helianthus annuus*, *Lactuca saligna*, *Lactuca sativa*, and the full-length transcripts of *Artemisia argyi* leaf were treated as database respectively, and *ADS* amino acid sequences of *Artemisia annua* were used as a query for blastp or tblastn (identity ≥ 40%, query coverage ≥ 80%).

Prediction and calculation of protein properties such as molecular weight and isoelectric point of *ADS* using ProtParam online tool (http://web.expasy.org/protparam/, accessed on 29 April 2022). And Cell-PLoc (http://www.csbio.sjtu. edu.cn/bioinf/Cell-PLoc-2/, accessed on 29 April 2022) was used to predict the subcellular localization of the identified *ADS* proteins to demonstrate the specific location of their presence within the cell.

### Phylogenetic analysis of ADS genes and similar genes

Multiple alignments of *A. annua ADS* protein sequences with protein sequences of similar genes of *C. cardunculus*, *H. annuus*, *L. saligna*, *L. sativa*, *C. nankingense, A. argyi* were performed by ClustalW of MEGA version 6 with the default parameter setting. The phylogenetic trees was built by MEGA version 6 by the Maximum likelihood (ML) or Neighbor joining method, and then the bootstrap values was set to 500 to achieve five hundred replicates.

For the analysis of the positive selection sites of the phylogenetic tree, EasyCodeML software, which is a CodeML visual analysis tool, was used [49]. The Site model was selected for analysis, and there were eight different hypothesis models in the Site model, in which M0 and M3, M1a and M2a, M7 and M8, and M8a and M8 were four pairs of Nested model, M0, M1a, M7 and M8a were Null model, and M3, M2a and M8 were Alternative model.

### Protein conserved motifs and gene structure analysis

The sequence and chromosome annotation data of *ADS* genes were downloaded from GPGD (http://www.gpgenome.com/, accessed on 10 May 2022). While *Cna4665*, *Cna4666*, and *Cna9606* were downloaded from Chrysanthemum Genome Database (http://www.amwayabrc.com/, accessed on 10 May 2022). The discovery of conserved motifs for these genes was attributed to the online MEME program (http://meme-suite.org/tools/meme, accessed on 11 May 2022). The parameter “how many motifs should MEME find” was set to 15, and leave the rest of the parameters unchanged at their default values. The NCBI CD -search Tool (https://www.ncbi.nlm.nih.gov/Structure/bwrpsb/bwrpsb.cgi/, accessed on 11 May 2022) was used to display their conserved domains. Besides, TBtools is a comprehensive bioinformatics tool that can be used to graphically show the motifs and structure of the above mentioned genes.

### Secondary structure, tertiary structure prediction and molecular docking of ADS gene and Cna4666

The secondary structure of *ADS* in *Artemisia annua* L. and *Cna4666* in *Chrysanthemum nankingense* were projected by an online program called SOPMA (https://npsa-prabi.ibcp.fr/cgi-bin/secpred_sopma.pl, accessed on 4 August 2022). And we used the online tool ColabFold [50], a tool combined the MMseqs2 with AlphaFold2 (https://colabresearch.google.com/github/deepmind/alphafold/blob/main/notebooks/AlphaFold.ipynb, accessed on 10 December 2022) to model the three-dimensional structural homology of the protein spatial model of *ADS, Cna4666* and proteins in phylogenetic tree. Then use AutoDock software to build a molecular docking model.

### Extract and analyze products of recombinant p0_ADS.1 and Cna4666 proteins using GC–MS analysis

Fllowing method draws on the earlier reaearch [22]. The coding sequences of the *Cna4666* (http://www.amwayabrc.com/) gene and *p0_ADS.1* (http://www.gpgenome.com/) were provided by TSINGKE Biological Technology Co., Ltd. (Beijing, China). Both genes were cloned into pET28a with BamHI and EcoRI enzyme cut sites. Then pET28a-p0_ADS.1 and pET28a-Cna4666 were transformed into E.coli BL21 (DE3) pLysS cells (TSINGKE Biotech Co., Ltd, Beijing, China). Cells grown at 37 °C and in 30 mL LB medium with 50 μg/mL Kanamycin, until OD_600_ reached 0.6–0.8. The induction of E. coli cells was achieved by adding 0.4 mM isopropyl-b-D-thiogalactoside (IPTG) to the bacterial solution and expressed it at 25 °C for 10 h. SPME Fiber (Merck KGaA, USA) were suspended on the bacterial solution in a 55 °C thermostatic equipment (water bath) for 30 min. Then the SPME Fiber was injected into a gas chromatograph injection unit.

In brief, the *p0_ADS.1* and *Cna4666* proteins was extracted using His- Tagged Protein Purification Kit (Soluble Protein, CWBIO, China). And sonication of the cells was carried out for 15 min in 5-s pulses with 5 s between pulses on ice, with the power set at 12 W. The system for the enzymatic reaction was to add 10 μg of protein to a mixture of Tris–HCl (pH = 8.0): 2-[4-(2-hydroxyethyl)piperazin-1- yl]ethanesulfonic acid at a final concentration of 30 mM, MgCl_2_ at a final concentration of 25 mM, dithiothreitol at a final concentration of 5 mM, and the final concentration of FPP was 60 μM. This system was placed in a glass sample vial covered with hexane and then reacted in a constant temperature water bath at 30 °C for 2 h. Then the hexane was injected into a gas chromatograph injection unit.

GC–MS analysis for product identification was performed by injecting a sample (split less mode) onto a WM-5MS column: 30 m × 0.25 μm × 0.25 mm. After an initial oven temperature of 250 °C during the injection period, the oven temperature was programmed from 50 °C (hold 5 min) to 260 °C (hold 3 min) at a rate of 8 °C/min. The carrier gas is helium, and flow rate was 1 mL/min. The column was connected to the ion source of a GCMS-QP2010 Ultra mass spectrometer, working in the 70-eV EI ionization mode and scanning from m/z 24 to 300.

### Chromosomal location and gene duplication analysis of ADS genes

The position information of *ADS* genes was acquired from the genomic sequence annotation and the package gggenes(R) was used to map gene locations. Gene duplication analysis was performed for *ADS* genes using the following strategy: (1) > 85% identity of the aligned region, (2) the coverage of coding sequence which is > 90% for potentially tandem duplicated genes (3) if there were less 100 kb and 5 or fewer than 5 genes separated by two *ADS* genes, they were labeled as tandem duplications.

### Selective pressure and synteny analysis of ADS and similar genes

We used Quick Run MCScanX Wrapper of TBtools to demonstrate the synteny relationship of the *ADS* orthologous genes present in Artemisia annua L. and *Helianthus annuus* or *Chrysanthemum nankingense*, the syntenic analysis plots were created by the Multiple Synteny Plot of TBtools [51, 52]. The KaKs Calculator 2.0 was used to calculate the Ks (synonymous) and Ka (non-synonymous) substitution for each *ADS* genes and similar genes.

## Result

### Identification of ADS genes in *A. annua* and similar genes in other species

Eight *ADS* genes (named *p0_ADS.1, p0_ADS.2, p0_ADS.3, p0_ADS.4, p1_ADS.5, p1_ADS.6, p1_ADS.7, p1_ADS.8*) were identified in the *A. annua* genome. Their identities between them ranged from 99 to 100%. The properties and subcellular localization of *ADS* genes were analyzed. The code lengths of these genes is 1641 bp and they encoded molecules of approximately 546 amino acids in length; their relative molecular masses ranged between 63.88kD (p0_ADS.4) and 63.97kD (p0_ADS.3) with a predicted isoelectric point (PI) range of 5.52 to 5.64, indicating that these *ADS* proteins were weakly acidic. Subcellular localization prediction showed that all 8 *ADS* paralogs were localized in chloroplast (Additional file 1: Table S1).

A total of 121 terpene synthase genes similar to *ADS* genes, with identity greater than 40% and query coverage greater than 80%, were identified in our Asteraceae plant genome dataset. There were 28 genes identified in the *A. annua* genome, 8 in *A. argyi* transcriptome, 8 in *C. cardunculus* genome, 23 in *H. annuus* genome, 13 in *L. saligna* genome, 12 in *L. sativa* genome, 29 in *C. nankingense* genome. These genes were renamed according to their species names and IDs, and all of them have terpene synthase family domain (pfam03936 and pfam01397) according to the analysis of the NCBI Batch CD-search online tool (Additional file 2: Table S2, Additional file 3: Table S3). And through MEME analysis, we can know that most of them have DDxxD and NSE/DTE motifs. By NCBI BLASTP analysis, some of them were identified as β-farnesene synthase, in addition to beta-caryophyllene synthase, (-)-germacrene D synthase, germacrene-A synthase, Epi-cedrol synthase (identity more than 80%).

### Phylogenetic analysis of *ADS* and *ADS*-like genes

**Fig. 1:**
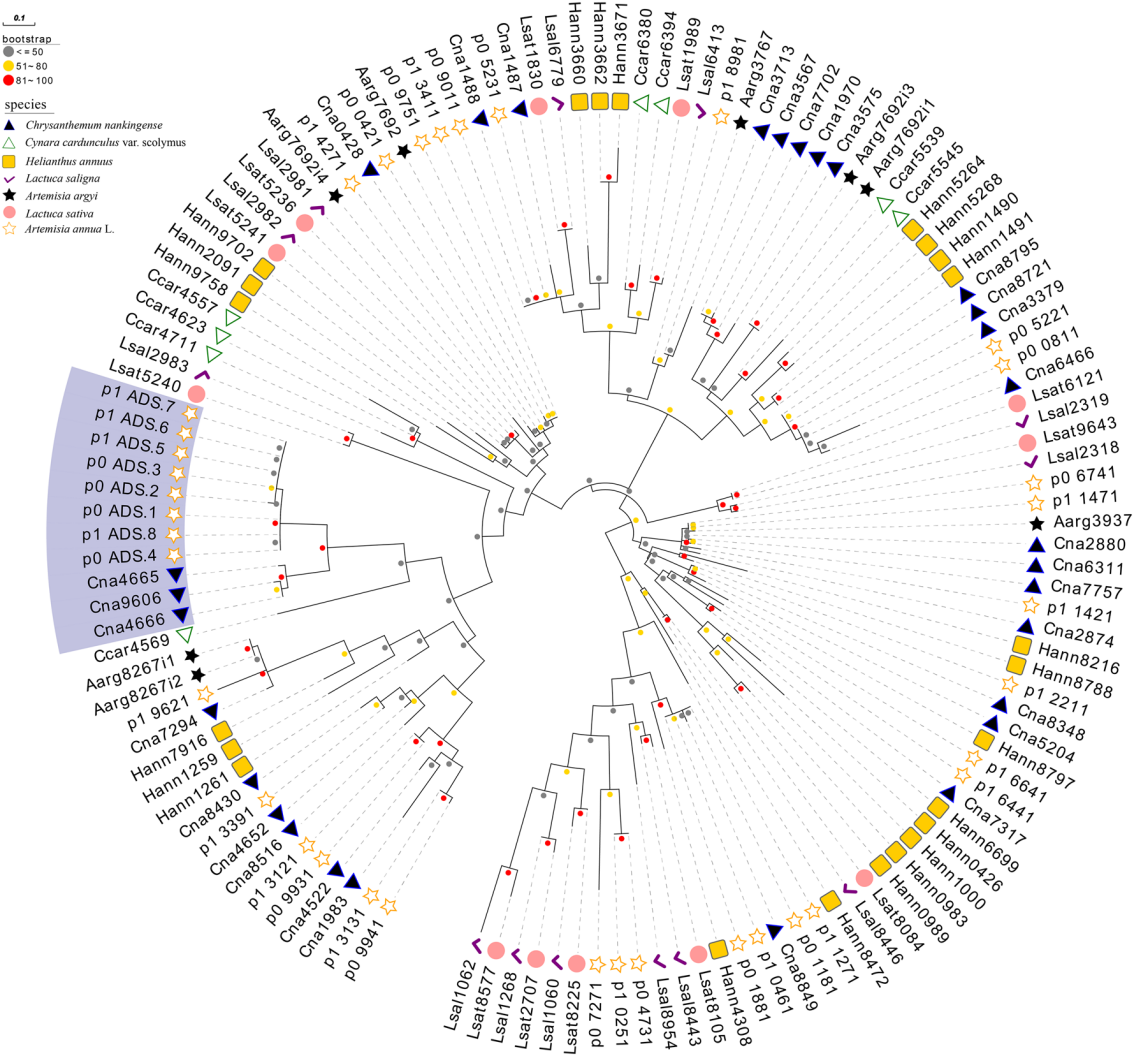
Phylogenetic relationship of *ADS* protein sequences of *A. annua* and its Homologous of related species. This phylogenetic tree uses the ML method, and the different colored circular dots of each node in the phylogenetic tree indicate different sizes of bootstrap, gray indicates ≤ 50, yellow indicates 51 ~ 80, and red indicates 81 ~ 100. Different colored shapes in front of the leaf labels indicate different species, please see the illustration for details

Based on the full-length *ADS* proteins sequences in *A. annua* (8), and similar proteins from the genome of *A. argyi* (8), *C. cardunculus* (8), *H. annuus* (23), *L. saligna* (13), *L. sativa* (12), *C. nankingense* (29), and *A. annua* (28), phylogenetic analysis were performed in a sequence alignment software, MEGA version 6, and a phylogenetic trees were built using the ML method (Fig. 1). The NJ method was then used to build phylogenetic trees to cross-validate the credibility of the clusters, the results show that the clusters of the two trees are basically the same (Additional file 5: Fig. S2). However, the clustering of the two trees resulted in different outgroups, so the conserved motif analysis of these 129 proteins proved that the outgrouping of the ML tree was more reasonable, since *Hann0426*, *Hann1000*, *Hann0983* and *Hann0989* have an additional characteristic motif (Additional file 6: Fig. S3). The ML tree showed that all *ADS* proteins from *A. annua* clustered in the same clades, *Can4665*, *Can4666*, and *Can9606* are clustered with *ADS* on one branch and sharing the same node with *Ccar4569*. This indicated that among all homologs, *Cna4665*, *Cna4666*, *Cna9606*, and *Ccar4569* are more closely related to *ADS*. Even they are more closely related to ADS genes than *ADS*-like genes in *A. annua*. While *Cna4665* and *Cna4666* have 82% identity with the *ADS* gene, *Cna9606* was 72%. This gives us reason to suspect that they have the potential to synthesize amorpha-4,11-diene. In *C. Cardunculus*, the more closely genes related *ADS* is *Ccar4569*, in *Helianthus annuus* are *Hann7916*, *Hann1259*, and *Hann1261*; in *L. saligna* is *Lsal2983*; in *L. sativa* is *Lsat5240*; in *A. argyi* are *Aarg8267i2* and *Aarg8267i1*. Furthermore, according to the result of NCBI BLASTP, *Ccar4569*, *Hann7916*, *Hann1259*, *Hann1261*, *Lsat5240*, *Aarg8267i2* and *Aarg8267i1* are more similar to beta-caryophyllene synthase [41–43].

In order to analyze which site play a role for adaptive evolution in this phylogenetic tree, positive selection was detected at the molecular level using CodeML. The site model was used to detect the positive selection effect of sites in nucleotide sequences, and the Likelihood ratio test (LRT) results after the test showed that the p-values of the three pairs of nested models, M1a vs. M2a, M7 vs. M8 and M8a vs. M8, were all smaller than 0.01, indicating that the alternative model was significantly better than the null hypothesis model, indicating the presence of positive selection effect (Additional file 7: Table S4). Further analysis using a Bayes empirical Bayes procedure identified eight sites (alignment positions 5, 14, 17, 18, 19, 20, 23 and 24) that are under positive selection with posterior probabilities ≥ 0.95, The specific site locations can be found in Table S5 (Additional file 8: Table S5). These results suggest that these eight sites may be factors that influence the function of these enzymes differently. In order to have more clarity on where these sites are located in the protein, we selected some representative proteins from the phylogenetic tree for 3D structure prediction (Additional file 9: Fig. S4). It turned out that these sites of *Last8084* in the outgroup are in the α-helix, while other proteins are in the random coil.

### Motif composition and gene structure of *ADS* genes and some similar genes

**Fig. 2:**
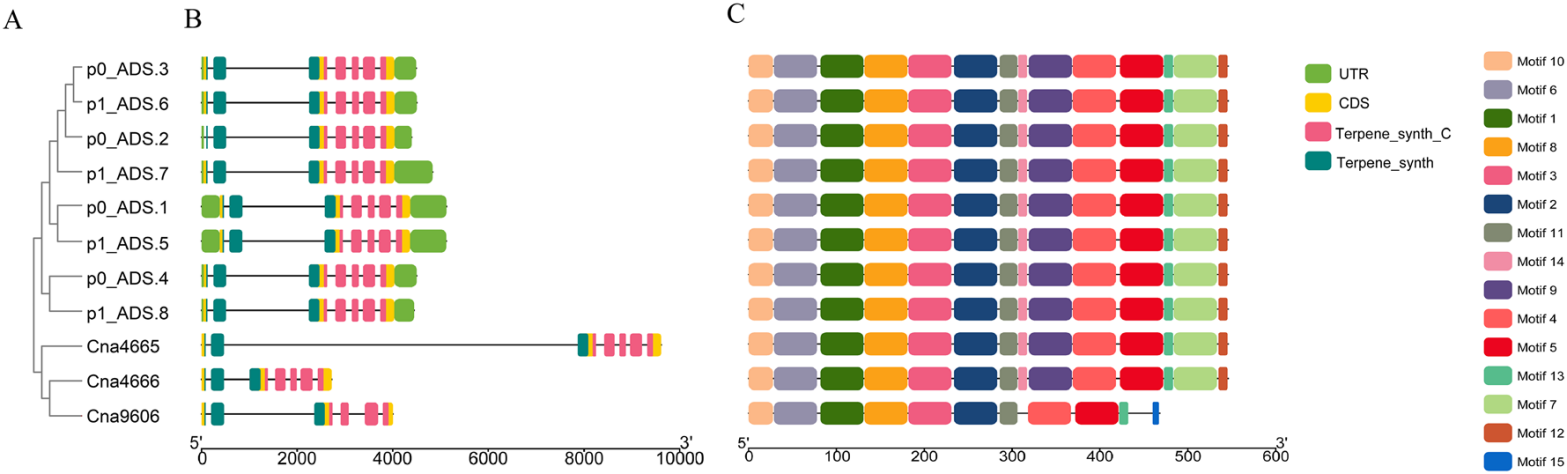
Gene structure, conserved motif, and genetic relationship of *ADS* and *Cna4665*, *Cna4666*, or *Cna9606*. **A** Phylogenetic analysis of *ADS* proteins and *Cna4665*, *Cna4666*, or *Cna9606*. **B** Exon/intron structure and conserved domains of *ADS* genes, *Cna4665*, *Cna4666*, and *Cna9606*. **C** The distribution of conserved motifs in *ADS* proteins, *Cna4665*, *Cna4666*, and *Cna9606*

To further investigate the structural peculiarities of genes in the same branch as *ADS* genes, the conserved motifs of proteins and intron/exon distributions of genes were analyzed (Fig. 2). According to the results, all *ADS* genes contain the same number of exons and introns, i.e. seven exons and six introns, and these exons and introns are of the same lengths. *Cna4665* and *Cna4666* also contain seven exons and six introns, but the second intron of *Cna4665* is longer than that of *ADS* genes, while *Cna4666* is shorter (Fig. 2B). Unlike the structure of the above genes, *Cna9606* possesses only six exons and five introns. Furthermore, MEME scanned a total of fifteen conserved motifs for these proteins, but not all of the proteins in the figure have these fifteen motifs. All of the *ADS* genes and *Cna4665*, *Cna4666* contain motif 1–14. *Cna9606* lacks motif 7, Motif 9, Motif 12, and Motif 14, but has an extra motif 15 in comparison with other genes (Fig. 2C). The results of all motif composition and gene structure analysis remind us that *Cna4666* and *Cna4665* are more likely to synthesize AD than *Cna9606*.

### Modeling of the secondary and tertiary structural homology of one *ADS* and Cna4666

**Fig. 3:**
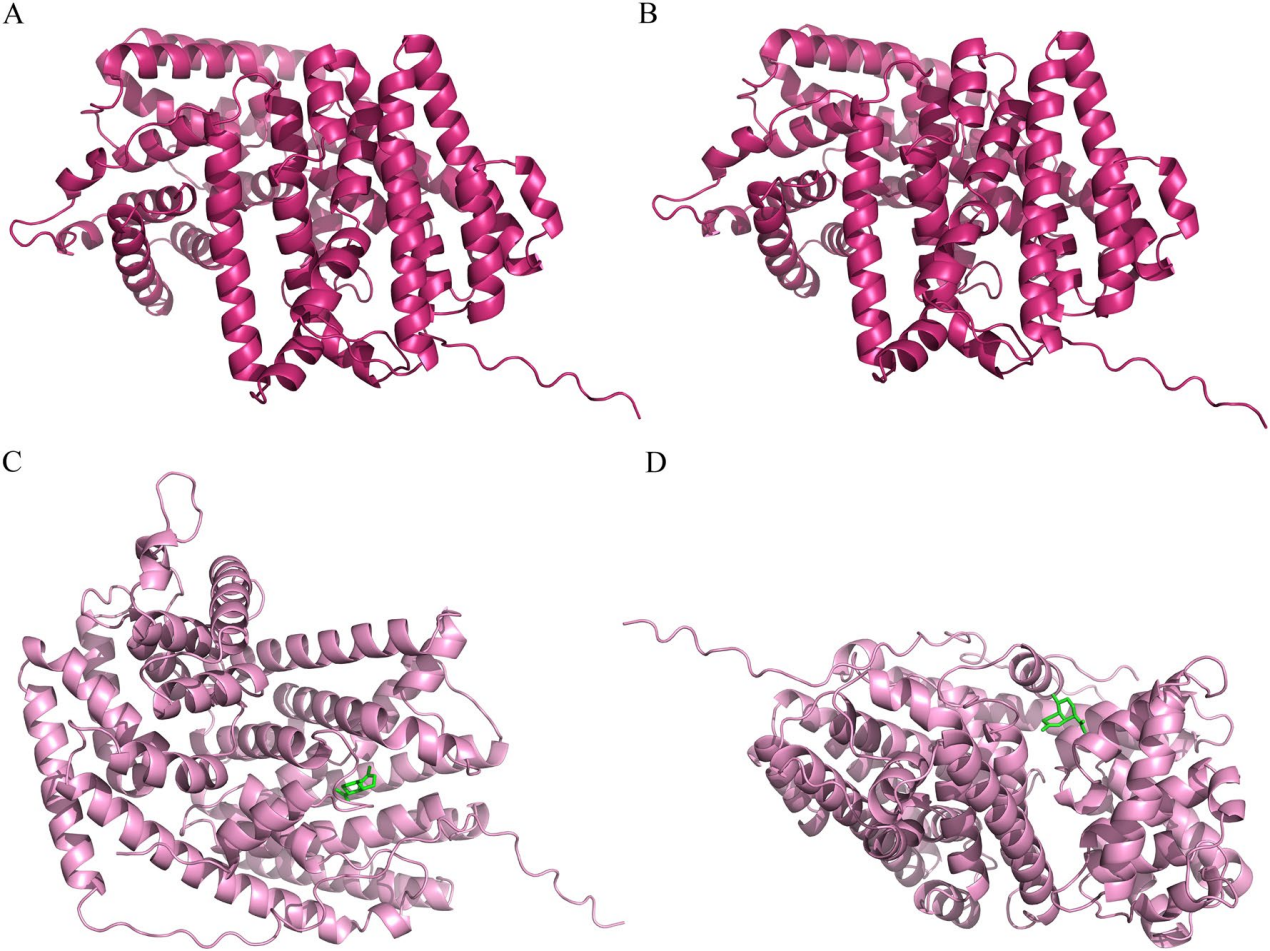
Tertiary structures and molecular docking model diagram of *p0_ADS.1* and *Cna4666* proteins. **A** Tertiary structures of *p0_ADS.1*. **B** Tertiary structures of *Cna4666*. **C** The *p0_ADS.1* molecular docking model with the highest absolute value of affinity. **D** The *Cna4666* molecular docking model with the highest absolute value of affinity

The secondary structure of *ADS* and *Cna4666* proteins were analyzed with SOPMA software. After sequence alignment, we learned that the DNA sequence similarity between *Cna4666* and *Cna4665* was 98.84%. and they are not more than 100 kb apart on the *C. nankingense* genome. They are a pair of tandem genes, so one of the two proteins was selected for prediction. The secondary structure of a protein refers to the regular spatial folding and coiling of the polypeptide chain backbone, which is determined by the hydrogen bonds between the non-side chain groups of amino acid residues, and it commonly includes α-helices, β-folds, random crimps, and β-turn. The results indicated that *p0_ADS.1* and *Cna4666* proteins share a very similar secondary structure (Table 1), and they have a higher percentage of α-helices (69.60% ~ 69.96%), a lower percentage of β-turn (2.75% ~ 3.30%), the same percentage of the extended strand (3.85%) and a slightly different proportions of the random coil (22.89% and 23.81%). The random coil is a flexible conformation that can change the direction of the peptide chain and facilitates the connection of relatively rigid α-helix and β-fold structure. It is speculated that *Cna4666* may have more variations in the orientation of polypeptide chains than *p0_ADS.1*. In addition, to initially compare their functions, their three-dimensional structures were predicted (Fig. 3A, B). According to the predicted results, the three-dimensional structures of *Cna4666 and p0_ADS.1* are extremely similar. Therefore, we used AutoDock to do the molecular docking of *ADS*, *Cna4666* with AD, and selected the highest absolute value of Affinity in the docking results (Fig. 3C, D). We found that the docking positions of the two proteins and AD were different, and the affinities were also different (− 7.3 and − 6.4, respectively), so we speculated that the function of *Cna4666* might be different from that of *ADS*.

**Table 1:** *p0_ADS.1* and *Cna4666* proteins secondary structure main component ratio

protein	α-helices	β-turn	Random coil	Extended strand
p0_ADS.1	69.96%	3.30%	22.89%	3.85%
Cna4666	69.60%	2.75%	23.81%	3.85%

### Functional characterization of *Cna4666* and *p0_ADS.1* using GC–MS

**Fig. 4:**
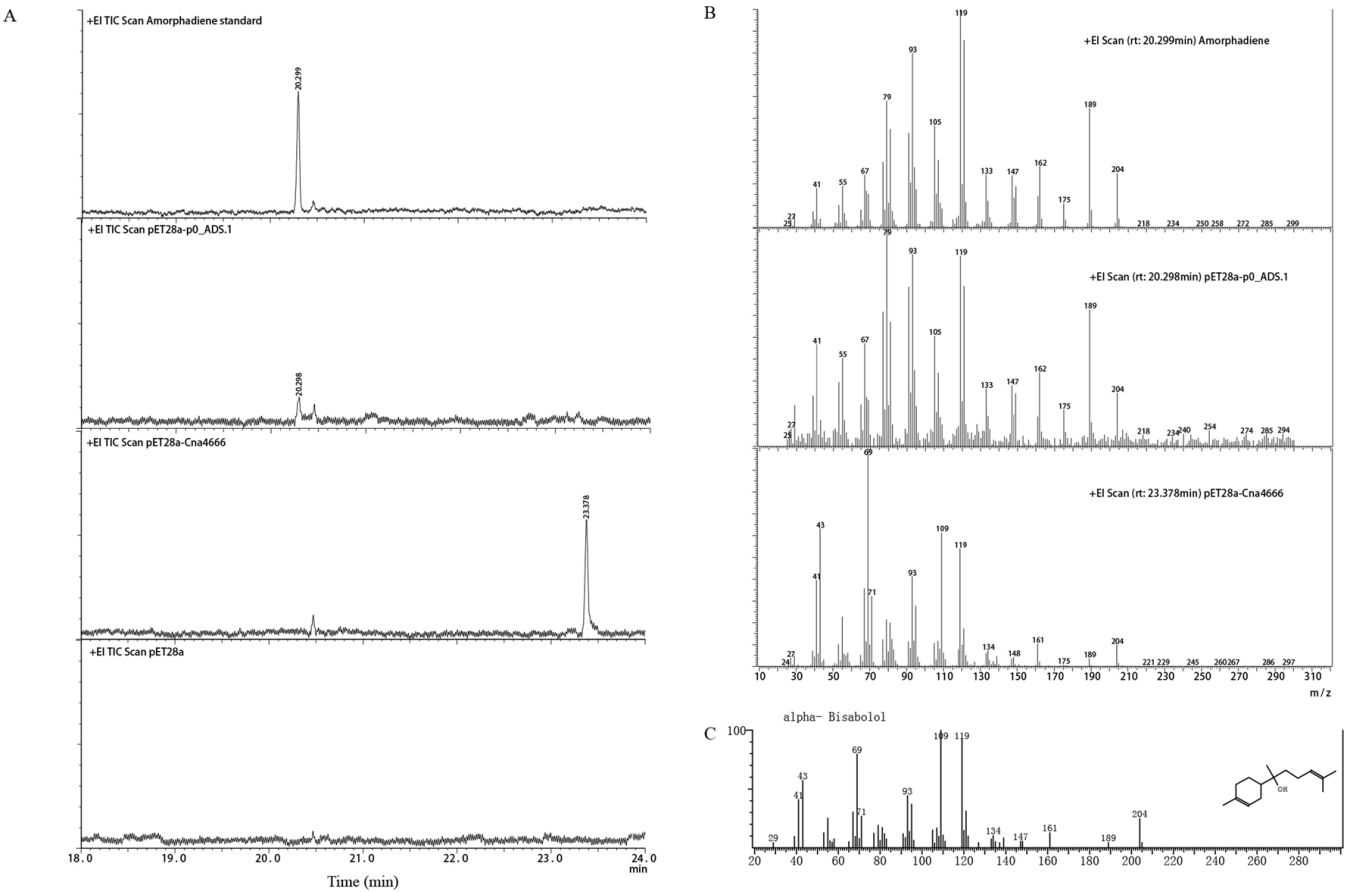
GC–MS analysis of the products formed by recombinant *Cna4666* and *p0_ADS.1* proteins. **A** Total ion current of products yielded by amorphadiene, pET28a-*p0_ADS.1*, pET28a-*Cna4666*, and pET28a control, respectively. **B** Mass spectrum of the indicated peak. **C** Mass spectrum of α-bisabolol

The precursors IPP and DMAPP of terpenoids in E. coli can be used to synthesize FPP, so E. coli has the precursor FPP of AD. The *Cna4666* and *p0_ADS.1* genes were cloned into a plasmid pET28a, and heterologously expressed in E. coli. The volatile products in the bacterial broth were then extracted using solid-phase microextraction for GC–MS analysis. The retention time of the expression product of the *Cna4666* gene was inconsistent with that of the expression product of *p0_ADS.1* gene (Fig. 4A); *p0_ADS.1* catalyzes the production of the AD, and the expression product of the *Cna4666* gene was characterized as α-bisabolol by mass spectrum (Fig. 4B, C). In addition, we successfully achieved an enzymatic reaction using the substrate FPP and the purified protein in combination with a suitable catalytic system, with the same catalytic results as in E. coli (Additional file 10: Fig. S5). These results indicates that *Cna4666* does not catalyze the production of amorpha-4,11-diene. And previous studies detected ( +)-α-bisabolol synthase in *A. maritima*, *A. kurramensis, A. abrotanum*, and *A. annua,* and showed that it does not synthesize AD, even in by-products [28, 53, 54]. All this reminds us that *ADS* may be present only in Artemisia, and that it is not widespread in Asteraceae.

### Gene duplication analysis and synteny analysis of *ADS* genes

**Fig. 5:**
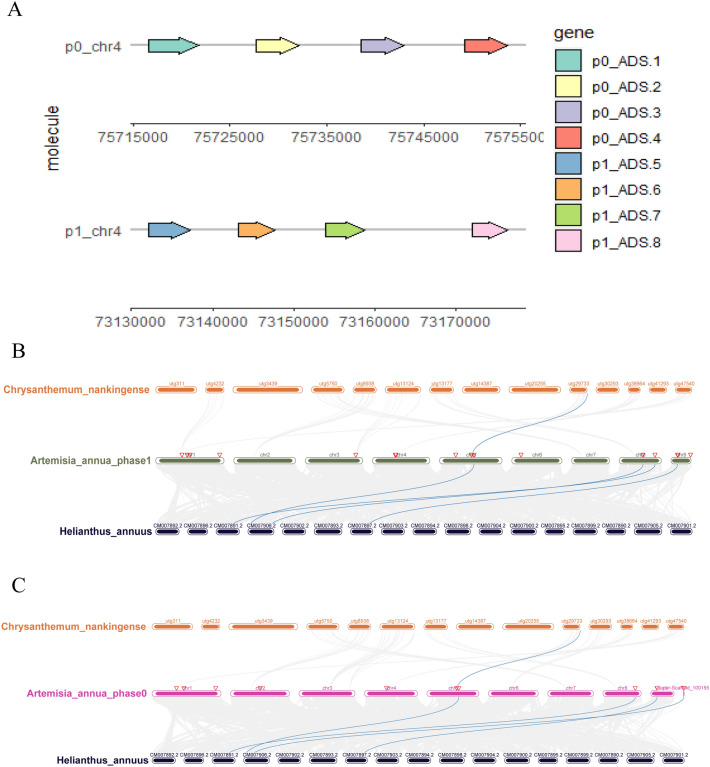
Gene duplication analysis of *ADS* genes and collinear association of *A.annua*, *H. annuus*, and *C. nankingense.* **A** The position of *ADS* genes in the two haplotype genomes. **B** Collinear association of *A. annua* haplotype1 (phase1), *H. annuus*, and *C. nankingense*. **C** Collinear association of *A. annua* haplotype0 (phase0), *H. annuus* and *C. nankingense*. Blue lines indicate collinear blocks of some *ADS* similar genes between *A. annua*, *H. annuus*, and *C. nankingense*

All of the *ADS* genes were evenly distributed on the Chromosomal 4 of the two haplotype genomes; *p0_ADS.1*, *p0_ADS.2*, *p0_ADS.3* and *p0_ADS.4* are alleles of *p1_ADS.5*, *p1_ADS.6*, *p1_ADS.7* and *p1_ADS.8*. The duplication events of the *ADS* genes were analyzed, because there were no other genes separated by two *ADS* genes and the maximum interval between two *ADS* genes is 4.5 kb, we can tell that these *ADS* genes are tandem duplicated genes (Fig. 5A). This also indicates that ADS is a multi-copy class of genes in the genome of *A. annua*.

We further constructed a synteny analysis between *ADS* genes in *A.annua* and homologous genes in other two plants, including *H.annuus* and *C.nankingense* to infer the evolutionary relationship between *ADS* and homologous genes among different species(Fig. 5B, C). We detected many syntenic orthologs in their genomes, but none of *ADS* genes and very few *ADS*-like genes were detected. There were 10 pairs of collinear blocks among the *ADS*-like genes, and the *ADS*-like genes *p0_5231*, *p0_1181*, *p0_7271*, *p0_5221*, and *p1_0461* of *A.annua* formed homologous pairs with *ADS*-like genes *Hann3660*, *Hann6699*, *Hann8472*, *Hann5264* of *H.annuus*. And the *ADS*-like gene *Cna1970* of *C.nankingense* found homologous gene pairs on chromosome 5 of two haplotype genomes of *A. annua*; *Hann3671*, *Hann8472* of H.annuus found homologous gene pairs on chromosome 5 and chromosome 8 of haplotype1(phase1) of *A. annua*, respectively. There was no colinear block of *ADS* genes in the *H.annuus* and *C.nankingense* genomes, and we hypothesize that *ADS* genes are recently evolved functional genes.

### Amino acid substitution selection pressure analysis of *ADS* and similar genes

Ka and Ks analysis was performed for *ADS* genes and ADS-like genes to estimate the selective pressure of these genes. The Ka/Ks ratio can show positive selection (Ka/Ks > 1), negative or purifying selection (Ka/Ks < 1), and neutral selection (Ka/Ks = 1) during evolution. The Ka/Ks values of *ADS* and these similar genes were lower than 1, indicating that purifying selection mainly contributed to the evolvement of these genes (Additional file 11: Fig. S6). In contrast, some of the Ka/Ks ratios within the *ADS* genes were much greater than 1 (Table 2), this result indicates that there is positive selection among the *ADS* genes and that they are undergoing rapid evolution, supporting the hypothesis that *ADS* is a recently evolved gene.

**Table 2:** Estimated Ka/Ks ratios of the *ADS* genes in *A. annua*

No	Sequence	Ka	Ks	Ka/Ks	Date (millions of years ago)
1	*p0_ADS.1*&*p0_ADS.2*	0.00298	0.00006	46.06050	0.0035
2	*p0_ADS.1*&*p0_ADS.3*	0.00314	0.00555	0.56649	0.3010
3	*p0_ADS.1*&*p0_ADS.4*	0.00393	0.00555	0.70743	0.3014
4	*p0_ADS.1*&*p1_ADS.5*	0.00142	0.00003	47.76480	0.0016
5	*p0_ADS.1*&*p1_ADS.6*	0.00223	0.00005	48.59900	0.0025
6	*p0_ADS.1*&*p1_ADS.7*	0.00235	0.00833	0.28257	0.4524
7	*p0_ADS.1*&*p1_ADS.8*	0.00314	0.00834	0.37674	0.4526
8	*p0_ADS.2*&*p0_ADS.3*	0.00157	0.00554	0.28312	0.3009
9	*p0_ADS.2*&*p0_ADS.4*	0.00393	0.00555	0.70785	0.3013
10	*p0_ADS.2*&*p1_ADS.5*	0.00155	0.00004	42.07890	0.0020
11	*p0_ADS.2*&*p1_ADS.6*	0.00072	0.00001	48.92730	0.0008
12	*p0_ADS.2*&*p1_ADS.7*	0.00078	0.00833	0.09415	0.4522
13	*p0_ADS.2*&*p1_ADS.8*	0.00314	0.00833	0.37696	0.4524
14	*p0_ADS.3*&*p0_ADS.4*	0.00393	0.01114	0.35261	0.6048
15	*p0_ADS.3*&*p1_ADS.5*	0.00195	0.00413	0.47162	0.2240
16	*p0_ADS.3*&*p1_ADS.6*	0.00078	0.00555	0.14140	0.3010
17	*p0_ADS.3*&*p1_ADS.7*	0.00078	0.00833	0.09415	0.4522
18	*p0_ADS.3*&*p1_ADS.8*	0.00314	0.01394	0.22533	0.7568
19	*p0_ADS.4*&*p1_ADS.5*	0.00393	0.00556	0.70659	0.3017
20	*p0_ADS.4*&*p1_ADS.6*	0.00314	0.00555	0.56565	0.3014
21	*p0_ADS.4*&*p1_ADS.7*	0.00314	0.01395	0.22513	0.7574
22	*p0_ADS.4*&*p1_ADS.8*	0.00078	0.00277	0.28291	0.1504
23	*p1_ADS.5*&*p1_ADS.6*	0.00083	0.00002	46.77650	0.0010
24	*p1_ADS.5*&*p1_ADS.7*	0.00078	0.00834	0.09398	0.4528
25	*p1_ADS.5*&*p1_ADS.8*	0.00314	0.00835	0.37629	0.4530
26	*p1_ADS.6*&*p1_ADS.7*	0.00001	0.01021	0.0010	0.5545
27	*p1_ADS.6*&*p1_ADS.8*	0.00243	0.00999	0.24309	0.5422
28	*p1_ADS.7*&*p1_ADS.8*	0.00252	0.02083	0.1209	1.1309

^1^Ka, non-synonymous rate

^2^Ks, synonymous substitution

^3^Ka/Ks, evolutionary constraint

The date of the duplication events (T) was calculated using the formula T = Ks/2λ (λ represents the estimated clock-like rate of synonymous substitution, which is 9.21E-9 substitutions/synonymous site/year for *A. annua*) [40]. The approximate dates of the estimated divergence time are shown in Table 2. The divergence time of *ADS* genes in *A. annua* ranged from 0.002 to 1.1309 MA.

## Discussion

Amorpha-4,11-diene synthase plays an important role in the synthesis pathway of artemisinin, and the gene of *ADS* was purified and functionally characterized for the first time in 1999 [6]. However, there are few reported studies on the relationship between *ADS* genes and *ADS*-like genes in other species. Therefore, in this study, we extracted *ADS* genes in the *A. annua* genome and *ADS*-like genes from the genomes of some Asteraceae, analyzed their molecular kinship, and explored whether *ADS* homologous genes exist in these Asteraceae plants.

In the present study, a total of 121 terpene synthase genes similar to *ADS* genes were identified. There were 28 *ADS*-like genes identified in *A. annua*, this number is lower than that in *C. nankingense* (29) but higher than that in *H. annuus* (23), *L. saligna* (13), *L. sativa* (12), *C. cardunculus* (8), *A. argyi* (8). Phylogenetic analysis revealed that *Cna4665*, *Cna4666*, and *Cna9606* are more closely related to *ADS* in these similar genes. Besides, *Cna4665*, *Cna4666*, and *Cna9606* are also the top three with the highest similarity to *ADS* sequences, with the similarity of 82.784%, 82.418%, and 72.179%, respectively. In the result of gene structural and conserved motif analysis, we found that *Cna4666* and *Cna4665* genes had very similar gene structures and the same motif as *ADS* genes. Based on the above results, we pointed out that *Cna4666* has high homology with *ADS*, and speculated that *Cna4666* may have the potential to synthesize AD. Meanwhile, we predicted the secondary and three-dimensional structures of *ADS* and *Cna4666*, and the results showed that they are very similar in both structures. So we then did a molecular docking analysis of the two proteins with AD, and the results showed that the docking models of AD and the two proteins were not the same.

In order to understand whether *Cna4666* has the potential to synthesize AD, we performed gene function identification. The results showed that *Cna4666* did not synthesize amorpha-4,11-diene, but synthesized another sesquiterpene, α-bisabolol, so *Cna4666* was identified as α-bisabolol synthase. This suggests that an enzyme such as ADS, which has a specific function in Artemisia, does not necessarily have the same function even if there are enzymes with extremely similar sequences and structures to it. This also indirectly proves that *ADS* genes are not widely present in Asteraceae. The expression of genes of the artemisinin synthesis pathway, including the *ADS* gene, has been reported in five species of Artemisia [46]. This suggests that *ADS* genes are present in some Artemisia plants. One genome of Artemisia, the *A. argyi*, was also recently published, but we did not have the opportunity to include *A. argyi* genome in our analysis due to insufficient data [55]. Some Artemisia plants have very important medical and economic values, and we hope that more Artemisia genomes will be published.

However, no collinear block of the *ADS* genes were found in the *Helianthus annuus* genome (a larger, more complete genome) and the *Chrysanthemum nankingense* genome(the genome in which *Cna4665* and *Cna4666* are present). Ka and Ks analysis demonstrates the selective pressure and divergence time of *ADS* genes, demonstrating that *ADS* genes are still being affected by positive selection pressure. Combining these two results, it is reasonable to speculate that *ADS* is a recently evolved gene in Artemisia. However, this conclusion still needs to be confirmed.

## Conclusion

Overall, this study reveals that *ADS* genes are inconsistency and not widespread in Asteraceae. It also explores the evolutionary trajectory of *ADS* in Asteraceae and draws the preliminary conclusion that *ADS* may be a recently evolved gene in the genus Artemisia. The exploration of *ADS* evolution is beneficial for a deeper understanding of artemisinin biosynthesis pathway genes, which can help in metabolic engineering of artemisinin to achieve the goal of high artemisinin production.

## Supplementary Information

**Additional file 1: Table S1.** Statistics of ADS gene information of *Artemisia annua* L.

**Additional file 2: Table S2.** Gene naming and characterization of other Asteraceae species.

**Additional file 3: Table S3.** Statistics of structural domains and gene families of all genes.

**Additional file 4: Figure S1.** The biosynthesis pathway for artemisinin. The orange route is the main synthesis path.

**Additional file 5: Figure S2.** Phylogenetic relationship of ADS protein sequences of A. annua and its Homologous of related species.

**Additional file 6: Figure S3.** Conserved motif and phylogenetic relationship of 129 genes extracted from the genome.

**Additional file 7: Table S4.** Results of CODEML analyses of selective pattern for ADS and ADS-like genes.

**Additional file 8: Table S5.** Sequence alignment data of *ADS* and *ADS*-like genes after removal of gaps.

**Additional file 9: Figure S4.** Tertiary structure of some representative proteins of the phylogenetic tree.

**Additional file 10: Figure S5.** GC–MS analysis of the products formed by recombinant Cna4666 and p0_ADS.1 proteins.

**Additional file 11: Figure S6.** KA and KS analysis of *ADS* genes and similar genes identified in Asteraceae plants.

## Data Availability

The datasets used and analysed during the current study are available from the corresponding author on reasonable request.

## Abbreviations

ADS: Amorpha-4,11-diene synthase
FP: Faresyl pyrophosphate
IPP: Isopentenyl pyrophosphate
DMAPP: Dimethylallyl pyrophosphate
MVA: Intracellular mevalonate
MEP: Methylerythritol 4-phosphate
FPS: Farnesyl pyrophosphate
AD: Amorpha-4,11-diene
CYP71AV1: Cytochrome P450 monooxygenase
ADH1: Alcohol dehydrogenase 1
DBR2: Double bond reductase
GPGD: Global pharmacopoeia genome database
NCBI: National Center for Biotechnology Information
SRA: Sequence read archive
ML: Maximum likelihood
