# Supplementary material for: Genetic analysis reveals the inconsistency of amorpha-4,11-diene synthase, a key enzyme in the artemisinin synthesis pathway, in asteraceae
Source: Chin Med. 2023 Jan 11;18:5. doi: 10.1186/s13020-023-00708-w (PMC9832723; doi:10.1186/s13020-023-00708-w)
Supplement: Supplementary file 4 — Additional file 4: Figure S1. The biosynthesis pathway for artemisinin. The orange route is the main synthesis path. [file 13020_2023_708_MOESM4_ESM.docx]

**Additional file 4: Figure S1.**


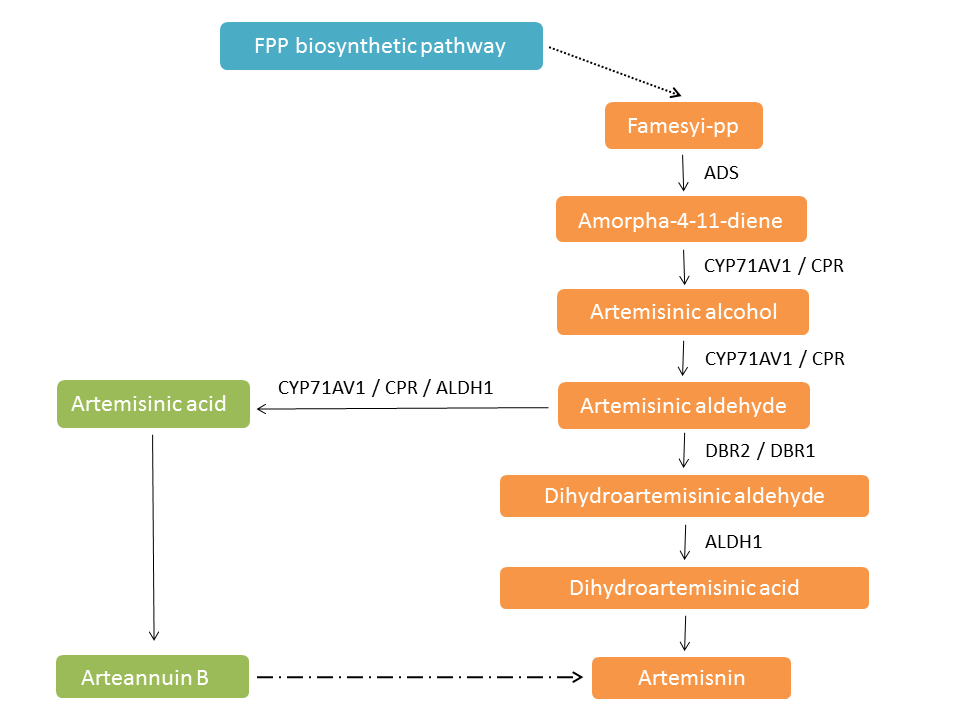


**Figure S1. The biosynthesis pathway for artemisinin.** The orange route is the main synthesis path.
