# Supplementary material for: Genetic analysis reveals the inconsistency of amorpha-4,11-diene synthase, a key enzyme in the artemisinin synthesis pathway, in asteraceae
Source: Chin Med. 2023 Jan 11;18:5. doi: 10.1186/s13020-023-00708-w (PMC9832723; doi:10.1186/s13020-023-00708-w)
Supplement: Supplementary file 5 — Additional file 5: Figure S2. Phylogenetic relationship of ADS protein sequences of A. annua and its Homologous of related species. [file 13020_2023_708_MOESM5_ESM.docx]

**Additional file 5: Figure S2.**


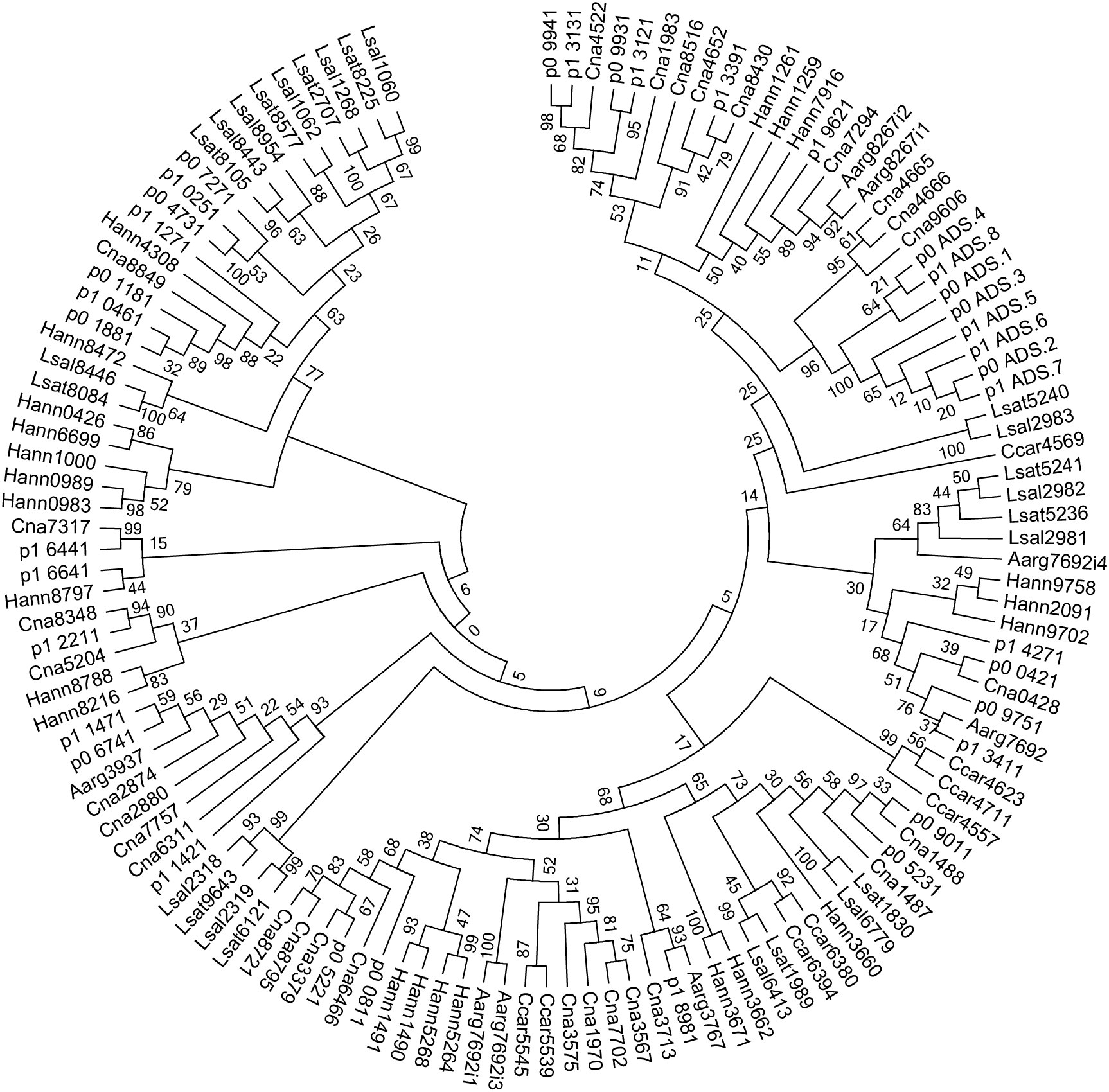


**Figure S2. Phylogenetic relationship of** ***ADS* protein sequences of *A. annua* and its Homologous of related species.** This phylogenetic tree uses the NJ method.
