# Supplementary material for: Genetic analysis reveals the inconsistency of amorpha-4,11-diene synthase, a key enzyme in the artemisinin synthesis pathway, in asteraceae
Source: Chin Med. 2023 Jan 11;18:5. doi: 10.1186/s13020-023-00708-w (PMC9832723; doi:10.1186/s13020-023-00708-w)
Supplement: Supplementary file 6 — Additional file 6: Figure S3. Conserved motif and phylogenetic relationship of 129 genes extracted from the genome. [file 13020_2023_708_MOESM6_ESM.docx]

**Additional file 6: Figure S3.**





**Figure S3. Conserved motif and phylogenetic relationship of 129 genes extracted from the genome.** A total of 50 motifs were identified in these genes.
