# Supplementary material for: Genetic analysis reveals the inconsistency of amorpha-4,11-diene synthase, a key enzyme in the artemisinin synthesis pathway, in asteraceae
Source: Chin Med. 2023 Jan 11;18:5. doi: 10.1186/s13020-023-00708-w (PMC9832723; doi:10.1186/s13020-023-00708-w)
Supplement: Supplementary file 9 — Additional file 9: Figure S4. Tertiary structure of some representative proteins of the phylogenetic tree. [file 13020_2023_708_MOESM9_ESM.docx]

**Additional file 9: Figure S4.**

**
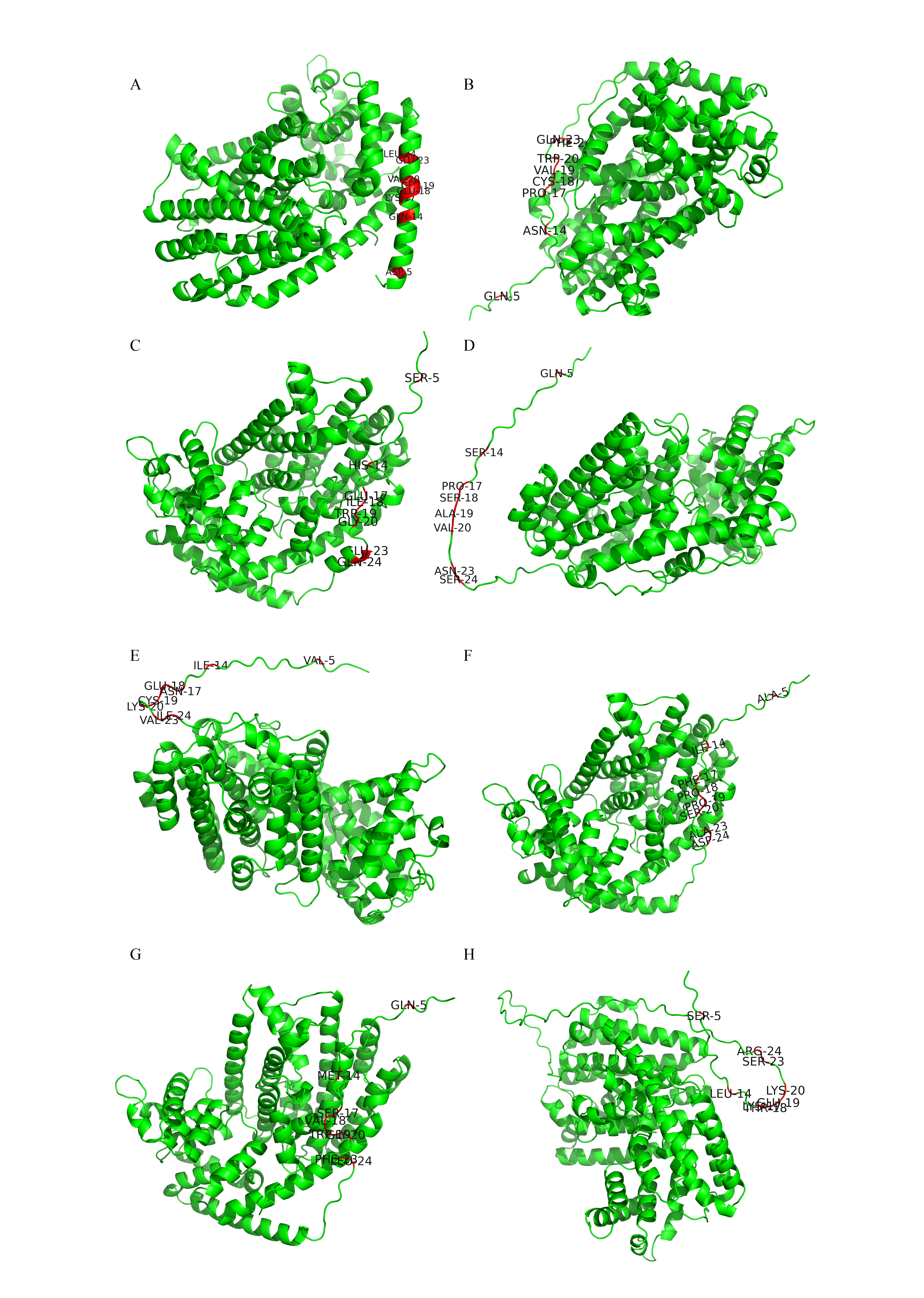
**

**Figure S4. Tertiary structure of some representative proteins of the phylogenetic tree.** (A) Tertiary structure of Last8084. (B) Tertiary structure of Hann6699. (C) Tertiary structure of Last9643. (D) Tertiary structure of Hann1491. (E) Tertiary structure of Ccar6380. (F) Tertiary structure of p1_3411. (G) Tertiary structure of Hann1261. (H) Tertiary structure of p0_9931.
