# Supplementary material for: Genetic analysis reveals the inconsistency of amorpha-4,11-diene synthase, a key enzyme in the artemisinin synthesis pathway, in asteraceae
Source: Chin Med. 2023 Jan 11;18:5. doi: 10.1186/s13020-023-00708-w (PMC9832723; doi:10.1186/s13020-023-00708-w)
Supplement: Supplementary file 10 — Additional file 10: Figure S5. GC–MS analysis of the products formed by recombinant Cna4666 and p0_ADS.1 proteins. [file 13020_2023_708_MOESM10_ESM.docx]

**Additional file 10: Figure S5.**


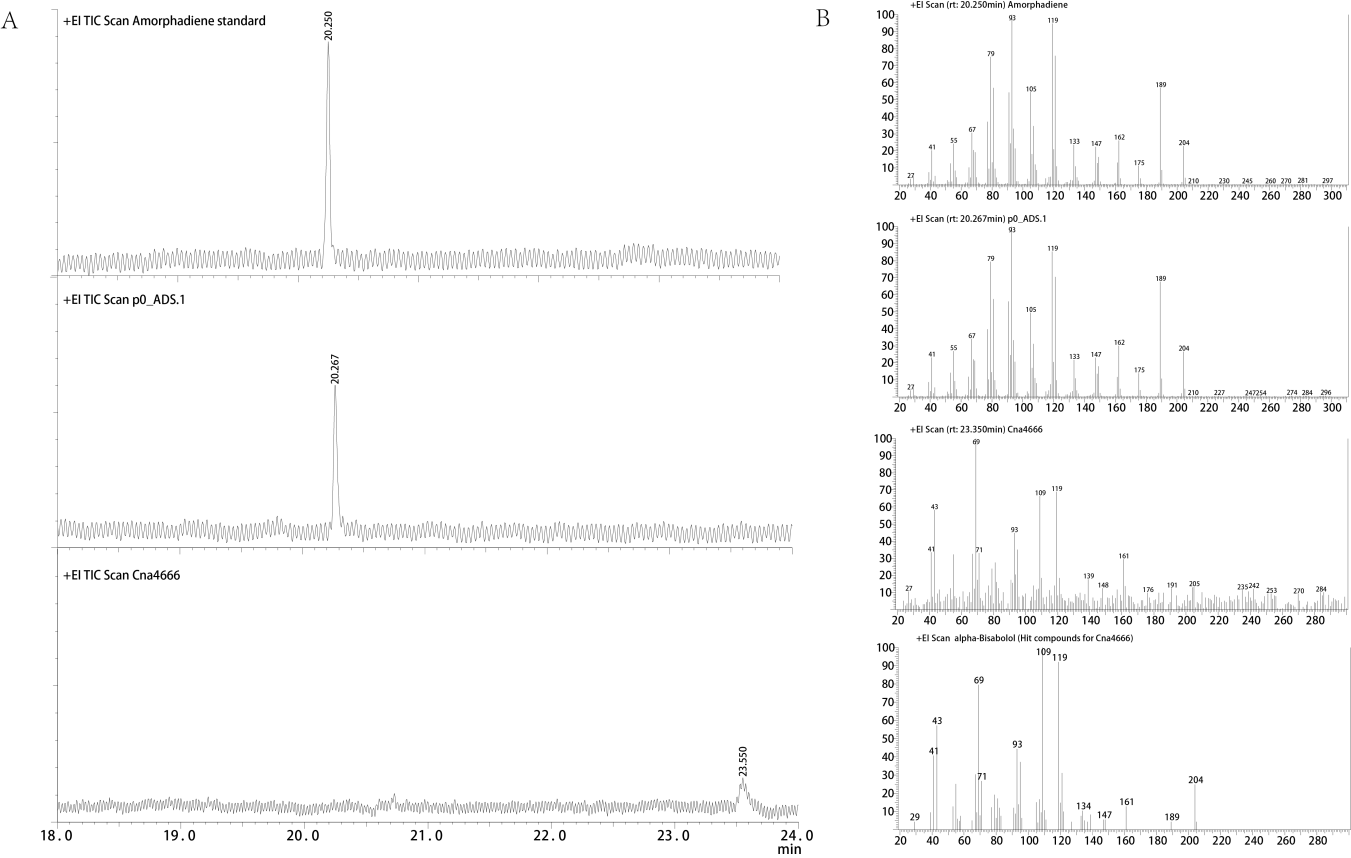


**Figure S5 GC-MS analysis of the products formed by recombinant *Cna4666* and p0_ADS.1 proteins.** **(A)** Total ion current of products yielded by amorphadiene, purified proteins of Cna4666, and p0_ADS.1, respectively. **(B)** Mass spectrum of the indicated peak.
