# Supplementary material for: Genetic analysis reveals the inconsistency of amorpha-4,11-diene synthase, a key enzyme in the artemisinin synthesis pathway, in asteraceae
Source: Chin Med. 2023 Jan 11;18:5. doi: 10.1186/s13020-023-00708-w (PMC9832723; doi:10.1186/s13020-023-00708-w)
Supplement: Supplementary file 11 — Additional file 11: Figure S6. KA and KS analysis of ADS genes and similar genes identified in Asteraceae plants. [file 13020_2023_708_MOESM11_ESM.docx]

**Additional file 11: Figure S6.**


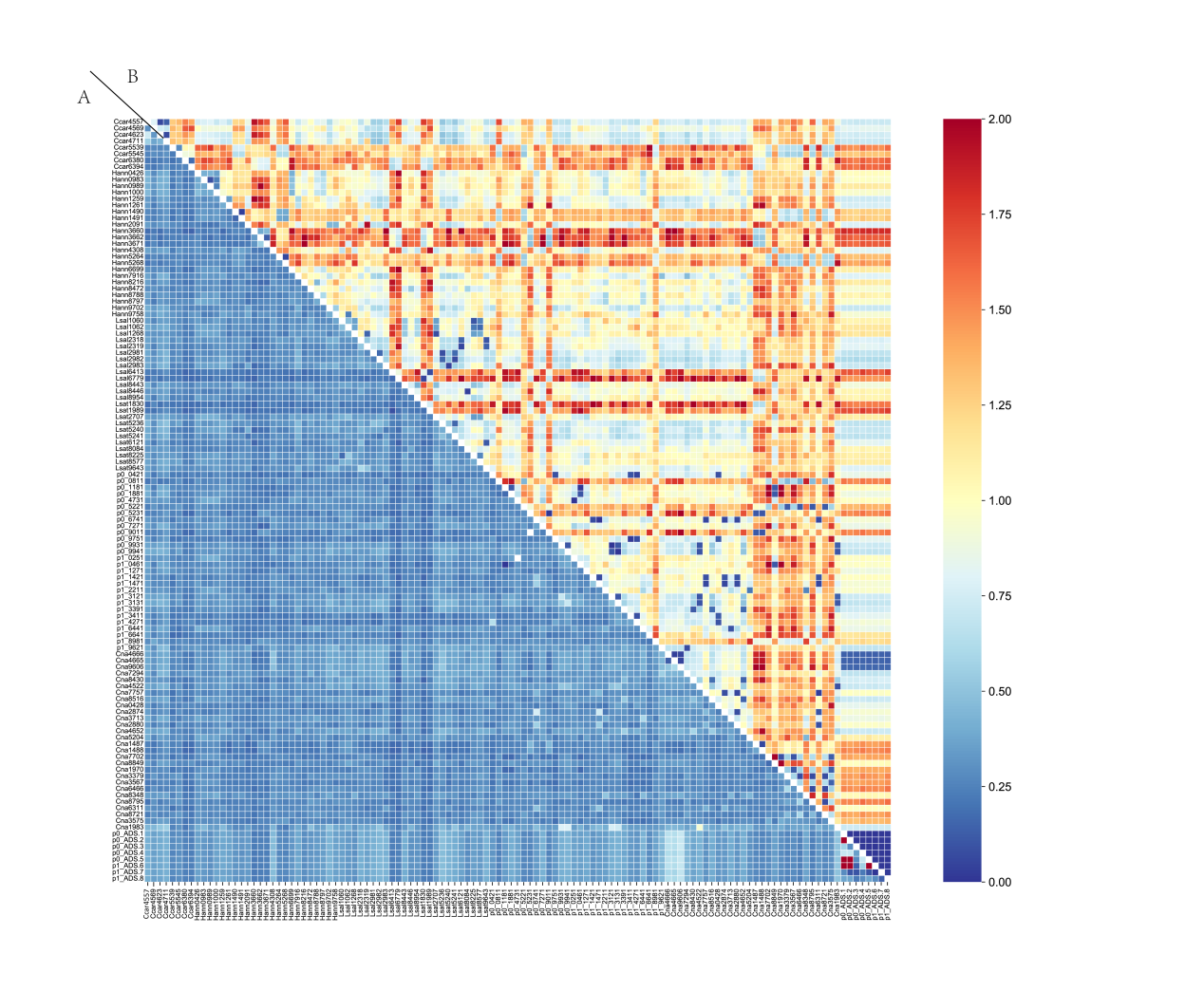


**Figure S6. KA and KS analysis of ADS genes and similar genes identified in Asteraceae plants. (A)** heat map showing the selective pressure of ADS genes and similar genes from 7 Asteraceae plants. **(B)** heat map showing the approximate divergence time of ADS genes and similar genes from 7 Asteraceae plants.
